# The effect of probiotic bacteria on toll-like receptor-2 and -4 expression by spermatozoa in couples with unexplained recurrent spontaneous abortion

**DOI:** 10.1016/j.bbrep.2022.101390

**Published:** 2022-12-05

**Authors:** Razieh Alipour, Nasrin Sereshki, Mitra Rafiee, Vahid Ahmadipanah, Davod Pashoutan Sarvar, Kourosh Rahimian, David Wilkinson

**Affiliations:** a Department of Immunology, School of Medicine, Isfahan University of Medical Sciences, Isfahan, Iran; b Department of Immunology, Cellular and Molecular Research Center, Birjand University of Medical Sciences, Birjand, Iran; c Asadabad School of Medical Sciences, Asadabad, Iran; d Medical Laboratory Sciences, Pasteur Clinical Laboratory, Sanandaj, Iran; e University of Aberdeen, Scotland, UK

**Keywords:** Abortion, Microbiota, TLR2, TLR4, Spermatozoa

## Abstract

The disturbance of microbiota composition in the female reproductive tract (FRT) can result in several reproductive disorders. Spermatozoa express toll like receptors (TLRs) and may encounter many types of microbiota in the FRT, however no study has been performed regarding the interaction between spermatozoa TLRs and FRT microbiota in unexplained recurrent spontaneous abortion (URSA) and fertile couples. In this study, we investigate the interaction of vaginal lactobacillus casei probiotic as a representative of FRT microbiota with TLR2 and 4 on spermatozoa. Ten fertile couples and ten URSA couples were involved in this study. Untreated and lactobacillus casei probiotic treated purified spermatozoa were evaluated for TLR2 and 4 expression by flow cytometry. Vaginal lactobacillus casei probiotic treatment of spermatozoa led to increased expression of TLR4 and decreased expression of TLR2 on spermatozoa in both URSA and fertile couples. Vaginal lactobacillus casei probiotic led to an increase in TLR4 expression and a decrease in TLR2 expression on spermatozoa in fertile and URSA groups. However, the disturbed expression of TLR2 and 4 was not completely correct, and further studies with other types of vaginal lactobacilli are needed. In contrast to our expectation, vaginal lactobacillus casei probiotic could not improve the disturbed expression of TLR2 and TLR4 in the RSA group. This could be due to small sample size and the use of one type of lactobacillus. Therefore, further study needs to be performed with other types of lactobacilli to determine the effect of microbiota and probiotics on spermatozoa function such as motility, acrosome reaction, sperm capacitation, sperm and egg fusion and spermatozoa motility and apoptosis and etc. Nevertheless, this study can provide a first step to investigate the effectiveness of vaginal microbiota on spermatozoa, and consequently design new strategies for RSA couples.

## Highlights

• The interaction of vaginal lactobacillus casei probiotic as a representative of FRT microbiota with TLR-2 & TLR-4 on spermatozoa.
• Probiotic treatment of spermatozoa led to increased expression of TLR-4 on spermatozoa in both URSA and fertile couples.
• Probiotic treatment of spermatozoa led to decreased expression of TLR-2 on spermatozoa in both URSA and fertile couples.

## Abbreviations

**FRT**: Female Reproductive Tract
**MRT**: Male Reproductive Tract
**URSA:** Unexplained Recurrent Spontaneous Abortion
**TLR**: Toll Like Receptor
**PRR**: Pattern Recognition Receptor
**PAMP**: Pathogen-Associated Molecular Pattern
**PE**: Phycoerythrin
**FITC**: Fluorescein-5-isothiocyanate
**AP-1**: Activator Protein-1
**IRF3**: Interferon Regulatory Factor 3

## 1 Introduction

Toll like receptors (TLRs) are a family of pattern recognition receptors (PRRs) that recognize pathogen-associated molecular patterns (PAMPs) derived from microorganisms, including pathogenic and nonpathogenic (such as commensal flora) organisms [1,2]. These receptors also recognize some endogenous ligands which are molecules derived from host tissues or cells, also components of cells or induced gene products in special situations [3]. Initiating signaling pathway of TLR through interaction with their ligands leads to activation of some transcription factors such as nuclear factor NF-κB and activator protein-1 (AP-1), interferon regulatory factor 3 (IRF3) and/or IRF7 [1,2,4].

Depending upon the type of TLR, the type of cell expressed TLR and the type of ligand, the activated transcription factor leads to the production of different proteins [1]. For example, activation of TLR5 on intestinal dendritic cells leads to IL-22 production and on intestinal epithelial cells controls the composition and localization of the intestinal commensal flora and prevention of intestinal inflammation [5]. TLRs are expressed by different cells including immune cells, such as lymphocytes and phagocytes, and non-immune cells, such as epithelial cells, endothelial cells, fibroblasts [2], spermatozoa [6,7] and cumulus cell-oocyte complex (COC) [8].

The TLRs play a critical role in both innate and adaptive immune responses [1,2,5]. Moreover, these receptors play some roles in reproduction [4,9]. As mentioned before, TLRs are expressed by human spermatozoa, although the role of these receptors in spermatozoa functions is not fully understood. However, an association between disturbed TLR4 and TLR2 expression on spermatozoa with unexplained recurrent spontaneous abortion (URSA) has been determined [10]. URSA is defined as three or more spontaneous abortions with unknown cause before 20–28 weeks gestation [11]. Other studies showed that TLR activation on spermatozoa results in decreased spermatozoa motility [12], induction of spermatozoa apoptosis [6] and impaired fertilization [12,13].

Spermatozoa encounter a large number of microbial organisms that are colonized in the female reproductive tract (FRT) and in the FRT formed commensal flora or microbiota. In most of the women, the predominant microbiota species in the FRT was lactobacilli. These bacteria have major roles in FRT health by mechanisms that include: a) reducing vaginal PH by the production of lactic acid, b) competing for cell surface and food, c) regulating the immune system, d) producing antimicrobial compounds, and e) enhancing the epithelial barrier function [14,15]. An imbalance in the composition of the FRT microbiota can result in reproductive disorders such as bacterial vaginosis, genital tract infection, infertility, early and late miscarriage, preterm delivery, postpartum endometritis and others [15].

The crucial role of lactobacilli in reproductive health has been the use of these bacteria as probiotics for treatment or complementary treatment of some reproductive diseases, such as bacterial vaginosis [[16], [17], [18]], urinary and genital tract infection [19,20] and preeclampsia [21,22]. Moreover, probiotics can have a beneficial effect on spermatozoa. An in vitro study showed that vaginal probiotic lactobacilli can prevent spermatozoa membrane lipid peroxidation and improve spermatozoa motility and viability [23].

In spite of many studies having been performed on the role of microbiota on reproduction health, there are very few studies in the context of FRT microbiota interaction with spermatozoa, specifically TLRs on spermatozoa. Given the association between URSA and the disturbed expression of TLR2 and TLR4 on spermatozoa, also the beneficial effect of probiotic on spermatozoa health, this study aims to determine the effect of vaginal probiotic lactobacillus on TLR2 and TLR4 expression by spermatozoa obtained from men with wives suffering from URSA.

## 2 Materials and methods

### 2.1 Subjects

Ten fertile couples with at least one child, as a control group, and ten URSA couples with no live birth, as a case group, were included in this study. URSA was diagnosed when other causes of RSA were ruled out, such as abnormalities of the uterus or cervix, chromosomal abnormality, infection, endocrine and metabolic diseases, congenital thrombophilia, autoimmune diseases and others. The husband of each woman in both groups had normal semen status, according to criteria from the World Health Organization (WHO 2010). All male partners did not have any history of genital tract disorder such as a history of infection, undescended testis, inguinoscrotal surgery, genital trauma or testicular torsion etc. Informed consent was obtained from all couples who participated in this study.

### 2.2 Purification of spermatozoa

Semen samples were collected after a 2–3 day period of sexual abstinence. Semen analysis testing was performed according to 2010 World Health Organization (WHO) criteria. Men who had abnormal quality semen were excluded from the study. Two gradient columns were prepared in a tube by gently layering 1 ml of AllGrad (LifeGlobal® Group, Canada) 90% gradient, followed by 1 ml of AllGrad 45% gradient. Then 1 ml of semen was added to this tube. The tube was centrifuged at 400 g for 18 min and then the pellet washed with AllGrad Wash. Finally, the pellet was re-suspended in Ham's F-10 medium (Dacell, Iran) with 1% bovine serum albumin (CMG, Iran).

### 2.3 Treatment of spermatozoa

Each purified spermatozoa sample was seeded in 4 wells of 96-well plates in volume 6 × 10^6^ cells/300 μl Ham's F-10 medium with 1% BSA (2 wells for probiotic treatment and 2 wells as untreated samples). One capsule of probiotic (Gynophilus vaginal capsules) containing lactobacillus casei rhamnosus Döderleini that is naturally present in the vaginal cavity was suspended in 1 ml Hams F-10 medium. Then 10 μl of this suspension was added to the wells that were specified for probiotic treatment. Cultures were incubated at 37 °C, 5% CO2 in a humidified atmosphere for 4 h.

### 2.4 Flow cytometric assay

Untreated and probiotic treated spermatozoa were stained with phycoerythrin (PE) mouse anti-human TLR4 (BD pharmingen, USA) and Fluorescein-5-isothiocyanate (FITC) mouse anti-human TLR2 (Southern Biotech). The density of cells was 1 × 10^6^ spermatozoa. After incubation at room temperature for 30 min and washing twice, spermatozoa were run through the flow cytometer (BD FACS Calibur, USA). Data from at least 100,000 events were collected using forward scatter (FSC) and side angle of light scatter (SSC). FSC data settings were linear and SSC and Fluorescence data settings were logarithmic. The FlowJo vx10 software was used for data analyses.

### 2.5 Statistical analyses

Two-factor mixed-design analysis of variance (ANOVA) for type (fertile, RSA), and treatment (untreated, probiotic treated) was conducted for TLR2 and TLR4. All statistical analyses were performed using IBM SPSS Statistics for Windows, version 20.0.0.1 (IBM Corp., Armonk, NY, USA). A P-value of <0.05 was considered significant. Type show between group and treatment within group.

## 3 Results

**Fig. 1:**
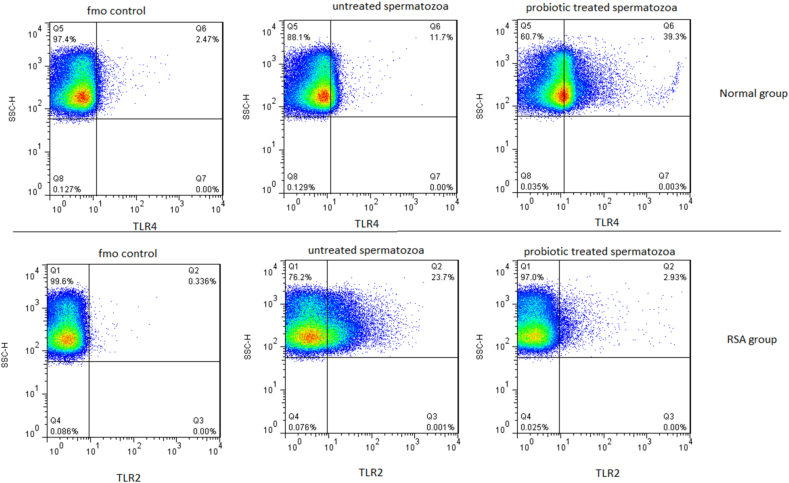
Flow cytometric analysis of TLR2 and TLR4 expression in Fluorescence Minus One (FMO) control, untreated and probiotic treated spermatozoa groups.

In this study, two variables (TLR2 and TLR4) were measured in two groups; firstly RSA (10 couples) and fertile (10 couples), and secondly untreated and probiotic treated spermatozoa. The TLR2 and TLR4 were measured by the flow cytometry method (Fig. 1).

The results of the Two-Way Mixed ANOVA analysis are as follows:

(1) The main effect between groups (fertile and RSA) for TLR2 was F(1,18) = 697.5, p < 0.001, ηp^2^ = 697.5 and for TLR4 was F(1,18) = 58.48, p < 0.001, ηp^2^ = 0.765. These results shows that the overall mean in the fertile group is significantly higher than in the RSA group (Table 1).

**Table 1:** Total mean of the TLR2 and TLR4 variables in the type groups.

Main effect			
**TLR2%**			
**Group**	Mean	SE	N
Fertile	33.21	1.37	10
RSA	24.85	1.37	10
**TLR4%**			
**Group**	Mean	SE	N
Fertile	31.27	1.24	10
RSA	17.77	1.24	10
(2) The main effect within groups (untreated and probiotic treated condition) for TLR2 was (F(1,48) = 45.6, p < 0.001, ηp^2^ = 0.717) and for TLR4 was (F(1,18) = 132.24, p < 0.001, ηp^2^ = 0.88). These results show that the overall mean of TLR4 in the probiotic treated condition is significantly higher than in the untreated condition, and conversely the TLR2 expression in probiotic treated spermatozoa is significantly lower than in untreated spermatozoa (Table 2).

**Table 2:** Total mean of the TLR2 and TLR4 variables in the treatment groups.

Main effect			
**TLR2%**			
**Treatment**	**Mean**	**SE**	**N**
Untreated	34.46	0.42	20
Probiotic treated	23.6	1.73	20
**TLR4%**			
**Treatment**	**Mean**	**SE**	**N**
Untreated	17.09	0.69	20
Probiotic treated	31.9	1.3	20

**Fig. 2:**
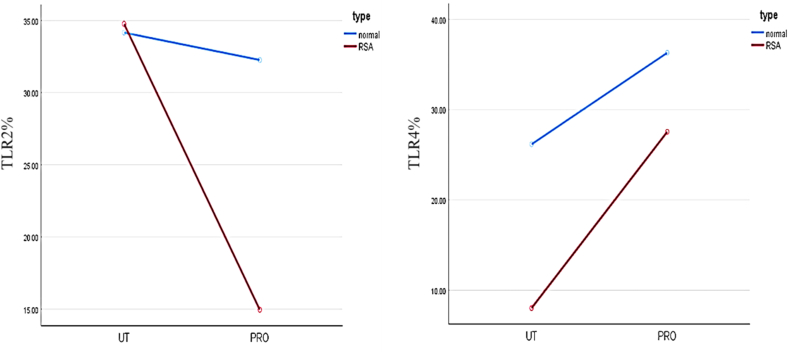
Profile plot of type (fertile, RSA) versus treatment (untreated [UT], probiotic treated [PRO]). Results show significant interaction between type (fertile, RSA) and probiotic treatment for TLR2 and TLR4.

Results for the impact of the probiotic treatment on spermatozoa in the fertile and RSA groups showed that there is a significant interaction between group (fertile or RSA) and probiotic treatment for TLR2 (F(1,18) = 31.07, p < 0.001, ηp^2^ = 0.63) and TLR4 (F(1,18) = 13.26, p < 0.001, ηp^2^ = 0.424). See Fig. 2.

A Bonferroni adjusted post hoc test was directed to indicate significant difference levels. In this test, the p-value was adjusted to 0.025 per test (0.05/2). For ease of reading, the results are shown in Table 3, Table 4. There were no significant differences in TLR2 between RSA and fertile groups, but probiotic treatment led to a decrease in TLR2 expression on spermatozoa in both the RSA and the fertile groups. In the case of TLR4, the level of this molecule on spermatozoa in the fertile group was higher than in the RSA group. Probiotic treatment of spermatozoa led to an increase in TLR4 on spermatozoa.

**Table 3:** Results obtained from treatment comparison (untreated, probiotic treated).

	Paired sample *t*-test				
		Untreated	Probiotic Treated	T, P, D	Explanation
**TLR2%**	Fertile	Mean = 34.16 Sd = 2.25 N = 10	Mean = 32.27 Sd = 1.4 N = 10	T(9) = 2.4 P = 0.41 D = 0.8	Non-significant
	RSA	Mean = 34.76 Sd = 1.4 N = 10	Mean = 14.95 Sd = 10.8 N = 10	T(9) = 6.3 P < 0.001 D = 2.01	Significant Probiotic < Untreated
**TLR4%**	Fertile	Mean = 26.16 Sd = 3.7 N = 10	Mean = 36.3 Sd = 2.6 N = 10	T(9) = -6.6 P < 0.001 D = 2.1	Significant Probiotic > Untreated
	RSA	Mean = 8 Sd = 2.3 N = 10	Mean = 27 Sd = 8.3 N = 10	T(9) = -9.4 P < 0.001 D = 2.8	Significant Probiotic > Untreated

RSA: Recurrent Spontaneous Abortion.

**Table 4:** Results obtained from the type comparison (fertile, RSA).

	Independent sample *t*-test				
		Fertile	RSA	T, P, D	Explanation
**TLR2%**	Untreated	Mean = 34.16 Sd = 2.25 N = 10	Mean = 34.76 Sd = 1.4 N = 10	T(18) = -0.7 P = 0.49 D = 0.32	Non-significant
	Probiotic Treated	Mean = 32.27 Sd = 1.4 N = 10	Mean = 14.95 Sd = 10.8 N = 10	T(9.3) = 4.9 P < 0.001 D = 2.24	Significant Fertile > RSA
**TLR4%**	Untreated	Mean = 26.16 Sd = 3.7 N = 10	Mean = 8 Sd = 2.3 N = 10	T(18) = 13.03 P < 0.001 D = 5.9	Significant Fertile > RSA
	Probiotic Treated	Mean = 36.3 Sd = 2.6 N = 10	Mean = 27 Sd = 8.3 N = 10	T(10.75) = 3.1 P = 0.005 D = 1.5	Significant Fertile > RSA

RSA: Recurrent Spontaneous Abortion.

## 4 Discussion

Human spermatozoa express different types of TLRs and, in the FRT encounter, a vast number of normal flora microbes (microbiota) that have a specific composition. We showed that probiotic contains lactobacillus casei rhamnosus Döderleini as a representative of FRT microbiota and leads to a decrease in TLR2 expression and an increase in TLR4 expression on spermatozoa in fertile and URSA couples. As far as we know, there is no study which investigates interaction of the FRT microbiota and spermatozoa TLRs in fertile and URSA couples.

In an earlier study, we showed that the expression of TLR2 differed between fertile and URSA groups (as in this study) while treatment of spermatozoa with PAM3CYS (a synthetic ligand for TLR2) lead to an increase in TLR2 expression in the URSA group and no significant differences in the fertile group [10]. In the present study, lactobacillus casei led to a decrease in expression of TLR2 in both groups, but the reduction in the level of TLR2 expression on spermatozoa in the RSA group was higher than in the fertile group. These results display an impaired ligand-TLR response in spermatozoa in the URSA group.

In the current, and also in our earlier, study we showed that the expression of TLR4 on spermatozoa from the RSA group is lower than from the fertile group. As mentioned above, probiotic treatment of spermatozoa led to an increase of TLR4 on spermatozoa in both groups. In our earlier study, LPS and PAM3CYS contrary to probiotic treatment could not make a significant change in TLR4 expression on spermatozoa. Results of this study may be evidence of the role of microbiota in spermatozoa function through interaction with spermatozoa TLRs.

Youko Fujita et al. showed that LPS and PAM3CYS in interaction with TLR4 and TLR2 on spermatozoa led to decreased motility and increased apoptosis of spermatozoa [6]. In Youko's study, LPS was derived from *E. coli* that is a pathogenic bacterium in the FRT. We hypothesized that microbiota in the male and female reproductive tract (MRT and FRT) probably have different effects on spermatozoa compared to pathogens and that the microbiota composition in the reproductive tract of URSA couples may differ from that of fertile couples.

Based on this hypothesis, we expected that vaginal lactobacilli probiotics could improve the disturbed microbiota composition and its adverse effect. Based on this hypothesis, we used commercially available vaginal lactobacillus casei for our study. However, we did not achieve the expected results. The cause of this could be due to two reasons; 1) small sample size, 2) we used only one type of vaginal lactobacillus, while spermatozoa encounter many different types of lactobacilli in the FRT. Therefore, further study needs to be performed with other types of lactobacilli to determine the effect of microbiota and probiotics on spermatozoa function such as motility, acrosome reaction, sperm capacitation, sperm and egg fusion and spermatozoa motility and apoptosis and etc.

## 5 Conclusion

This study showed that the vaginal microbiota lactobacillus casei, as a representative of FRT microbiota, has the ability to increase TLR4 expression on spermatozoa in RSA and fertile groups, however TLR2 expression decreased in both groups. In contrast to our expectation, lactobacillus casei could not improve the disturbed expression of TLR2 and TLR4 in the RSA group. This could be due to small sample size and the use of one type of lactobacillus.

Therefore, further study needs to be performed with other types of lactobacilli to determine the effect of microbiota and probiotics on spermatozoa function such as motility, acrosome reaction, sperm capacitation, sperm and egg fusion and spermatozoa motility and apoptosis and etc.

Nevertheless, this study can provide a first step to investigate the effectiveness of vaginal microbiota on spermatozoa, and consequently design new strategies for RSA couples.

## Ethics approval and consent to participate

The protocol of this study was performed in accordance with the relevant guidelines approved by the Ethics Committee of Asadabad School of Medical Sciences (Approval Number: IR.ASAUMS.REC.1399.001). Informed consent was obtained from all couples who participated in this study.

## Consent for publication

Not applicable.

## Availability of data and materials

The datasets generated during and/or analyzed during the current study are available from the corresponding author on reasonable request.

## Funding

This work did not receive any specific grant from funding agencies in the public, commercial or not-for-profit sectors.

## Authors' contributions

Nasrin Sereshki contributed to conception and design, experimental work, data and statistical analysis, interpretation of data, manuscript writing and editing. Razieh Alipour, Mitra Rafiee and Davod Pashoutan Sarvar contributed to all experimental work and interpretation of data. Vahid Ahmadipanah contributed data and statistical analysis. David Wilkinson contributed to critical editing of the manuscript. All authors read and approved the final manuscript.

## Declaration of competing interest

The authors declare that there is no conflict of interest regarding the publication of this article.
